# Machine learning for the prediction of 1-year mortality in patients with sepsis-associated acute kidney injury

**DOI:** 10.1186/s12911-024-02583-3

**Published:** 2024-07-25

**Authors:** Le Li, Jingyuan Guan, Xi Peng, Likun Zhou, Zhuxin Zhang, Ligang Ding, Lihui Zheng, Lingmin Wu, Zhicheng Hu, Limin Liu, Yan Yao

**Affiliations:** Fuwai Hospital, National Center for Cardiovascular Diseases, Chinese Academy of Medical Sciences, Peking Union Medical College, Beijing, 100730 China

**Keywords:** Sepsis, Acute kidney injury, Machine learning, Prediction model, Prognosis

## Abstract

**Introduction:**

Sepsis-associated acute kidney injury (SA-AKI) is strongly associated with poor prognosis. We aimed to build a machine learning (ML)-based clinical model to predict 1-year mortality in patients with SA-AKI.

**Methods:**

Six ML algorithms were included to perform model fitting. Feature selection was based on the feature importance evaluated by the SHapley Additive exPlanations (SHAP) values. Area under the receiver operating characteristic curve (AUROC) was used to evaluate the discriminatory ability of the prediction model. Calibration curve and Brier score were employed to assess the calibrated ability. Our ML-based prediction models were validated both internally and externally.

**Results:**

A total of 12,750 patients with SA-AKI and 55 features were included to build the prediction models. We identified the top 10 predictors including age, ICU stay and GCS score based on the feature importance. Among the six ML algorithms, the CatBoost showed the best prediction performance with an AUROC of 0.813 and Brier score of 0.119. In the external validation set, the predictive value remained favorable (AUROC = 0.784).

**Conclusion:**

In this study, we developed and validated a ML-based prediction model based on 10 commonly used clinical features which could accurately and early identify the individuals at high-risk of long-term mortality in patients with SA-AKI.

**Supplementary Information:**

The online version contains supplementary material available at 10.1186/s12911-024-02583-3.

## Introduction

Sepsis is a life-threatening clinical syndrome characterized by organ dysfunction caused by a patient’s dysregulated response to infection [1]. Acute kidney disease (AKI) is a syndrome defined as a fast increase serum creatinine (CRE), a decrease urine output (UO) or both [2]. Sepsis is highly correlated with AKI. In patients with sepsis, the kidneys are the most common organs to be affected. Sepsis is associated with up to 50% of AKI, and up to 60% of patients with sepsis have AKI [3, 4]. Moreover, sepsis-associated acute kidney injury (SA-AKI) is strongly associated with poor prognosis. Previous studies have demonstrated that SA-AKI was associated with higher risk of short- and long-term mortality, longer hospital stay and renal replacement therapy (RRT) requirement [5–7].

It is nearly impossible to identify the exact onset of AKI because of the complex and unique pathophysiology mechanism of sepsis, making it difficult to perform timely intervention for prevention of renal injury [8]. Accordingly, severity scores and risk stratification are the key points in the management of AKI, which is conducive to clinical decision-making. The current prediction models for mortality are limited by small sample size and unsatisfactory prediction performance [9, 10].

In recent years, machine learning (ML), which integrates mathematics and computer science, has been introduced in medicine prediction issues and presented with favorable prediction performance [11–14]. In this study, we aimed to establish a prediction model to early identify individuals at high-risk of long-term mortality in patients with SA-AKI, which may help to take appropriate preventive strategies and significantly improve outcomes for them.

## Methods

### Source of data

The patients’ data were obtained from two sources: the Medical Information Mart for Intensive Care IV (MIMIC-IV, version 2.0) and the MIMIC-III (version 1.4). The MIMIC-IV is a comprehensive US-based database that includes information from over 200,000 individuals who were admitted to various ICUs at the Beth Israel Deaconess Medical Center (BIDMC) between 2008 and 2019 [15]. On the other hand, the MIMIC-III database comprises data collected from the same hospital but during a different period compared to the MIMIC-IV database [16]. Therefore, data from the MIMIC-III database were utilized for temporal external validation purposes. Since this study involved the analysis of third-party databases with pre-existing institutional review board approval, ethical approval and consent to participate were not applicable. However, it is important to note that one of the authors has completed the Collaborative Institutional Training Initiative course and possesses the necessary certification (certification number 35,965,741) to access the databases. The study adhered to the recommendations outlined in the Transparent Reporting of a multivariable prediction model for Individual Prognosis Or Diagnosis (TRIPOD) statement [17].

### Study population

In this retrospective study, patients with sepsis who suffered from AKI during hospitalization were eligible for inclusion. In the present study, sepsis was diagnosed based on the Sepsis-3 criteria [18]. Moreover, AKI was diagnosed based on the following clinical practice guidelines: increase in CRE ( by ≥ 0.3 mg/dL (or ≥ 26.5 µmol/L) in 48 h, increase in CRE to 1.5 times over baseline levels in 7 days, and patient UO ≤ 0.5 mL/kg/h for 6 h [2]. The definition of SA-AKI was based on the consensus report of the 28th Acute Disease Quality Initiative workgroup. According to this report, SA-AKI should be considered when AKI occurs within 7 days of sepsis diagnosis, and can be further differentiated into early (AKI occurs up to 48 h after sepsis diagnosis) or late SA-AKI (AKI occurs between 48 h and 7 days of sepsis diagnosis) [19, 20]. Patients aged < 18 years old or length of stay in hospital < 48 h were excluded. The primary outcome was 1-year mortality after hospital admission.

### Data collection and imputation

We extracted a range of data from the two databases, including demographics, vital signs, laboratory test results, and comorbidities. To enhance the practicality of the model, we specifically chose the data from the first medical records rather than relying on the maximum or minimum values observed during hospitalization. The baseline creatinine levels are determined by the results of the first biochemical blood test conducted within 24 h of the patient’s admission. During the data collection process, we encountered several variables with missing values. To address this issue, we implemented a systematic approach. Firstly, variables with a missing value ratio exceeding 30% were excluded from the analysis. For variables with missing values below 5%, we utilized mean imputation to impute the missing data. Additionally, for features with missing values ranging from 5 to 30%, we employed multiple imputations to impute the missing data [21]. By utilizing these techniques, we aimed to minimize the impact of missing data on the accuracy and reliability of our findings.

### Model development and validation

#### Feature selection

We employed the Shapley Additive explanations (SHAP) values, a game theoretic approach, to assess the importance of each feature in our model [22]. This analysis allowed us to identify the key features that significantly contribute to the predictive performance. To enhance the practicality of the model and simplify its implementation, we selected the top 10 predictors as the main features for model building. By focusing on these highly influential predictors, we aimed to create a more efficient and user-friendly model that can effectively capture the essential information needed for accurate predictions.

#### Model evaluation

We utilized the area under the receiver operating characteristic curve (AUROC) to evaluate the discriminatory ability of the models. This metric provides a comprehensive assessment of the models’ ability to distinguish between positive and negative outcomes. To further evaluate the calibration of the models, we employed the calibration curve and the Brier score. The calibration curve offers a qualitative assessment of how well the predicted probabilities align with the observed outcomes, while the Brier score quantitatively measures the accuracy of the predicted probabilities. To assess the clinical utility of the models, we conducted decision curve analysis (DCA) to calculate the decision benefit. This analysis helps determine the net benefit of using the models in clinical decision-making. Furthermore, we evaluated the prediction performance of each model using various metrics, including accuracy, sensitivity, specificity, positive prediction value (PPV), negative prediction value (NPV), Matthews correlation coefficient (MCC), and F1-score. These metrics provide a quantitative evaluation of the models’ performance in terms of accuracy, true positive rate, true negative rate, precision, and overall predictive power.

#### Algorithm selection

We employed six commonly used machine learning algorithms, namely CatBoost, XGBoost, LightGBM, logistic regression (LR), random forest (RF), and Bagging, for model fitting. Each algorithm was evaluated based on its discriminatory and calibrated abilities. After evaluating the performance of these algorithms, we selected the one that demonstrated the best prediction performance for further analyses. This selection was based on the algorithm’s ability to achieve optimal discriminatory and calibrated results.

#### Model optimization

Hyperparameter optimization (HPO) was performed to optimize the prediction models. By finding the ideal combination of hyperparameters, the predictive performance of machine learning models can be significantly improved.To facilitate the visualization of the HPO process, we utilized the Optuna package (version 2.10.0), an open-source optimization framework. Optuna allowed us to efficiently and dynamically conduct HPO experiments by testing various combinations of hyperparameters. Specifically, we employed the Hyperband method within Optuna to perform HPO and identify the best set of hyperparameters for our models [23, 24].

#### Model validation

In this study, both internal and external validations were conducted to assess the robustness and generalizability of the model. For the external validation, data from the MIMIC-III database were utilized. Furthermore, we compared the predictive performance of the ML-based models with commonly used clinical scores such as the Logistic Organ Dysfunction System (LODS), Simplified Acute Physiology Score-II (SAPS), and Charlson comorbidity index. This comparison aimed to further demonstrate the superior predictive value of the ML-based models. To provide a visual representation of the prediction results, we developed a nomogram based on the external validation set. This nomogram allowed for a graphical presentation of the predicted probabilities. Additionally, a decision tree was constructed using recursive partitioning analysis, using the total points from the nomogram for risk stratification of patients. Furthermore, the predictions for each patient were plotted in order of their risk, providing an assessment of the prediction distribution generated by the model.

### Statistical analysis

All statistical analyses were conducted using Python (version 3.9.0) and R (version 4.1.0). The primary Python packages utilized in this study include *‘sklearn.model_selection’*, *‘catboost’*, *‘numpy’*, *‘pandas’*, *‘sklearn.metrics’*, and *‘shap’*, etc. Continuous variables were presented as mean ± standard deviation, while categorical variables were reported as numbers with percentages. To assess differences between two groups for continuous variables, the t-test was employed, while the chi-square test was used for categorical variables. Additionally, multicollinearity among the variables in the nomogram was evaluated using the variance inflation factor (VIF), where a VIF > 4.0 indicated the presence of multicollinearity. Logistic regression analyses were performed to identify the key factors among the included features. A significance level of *p* < 0.05 was considered statistically significant.

## Results

### Baseline characteristics

**Fig. 1:**
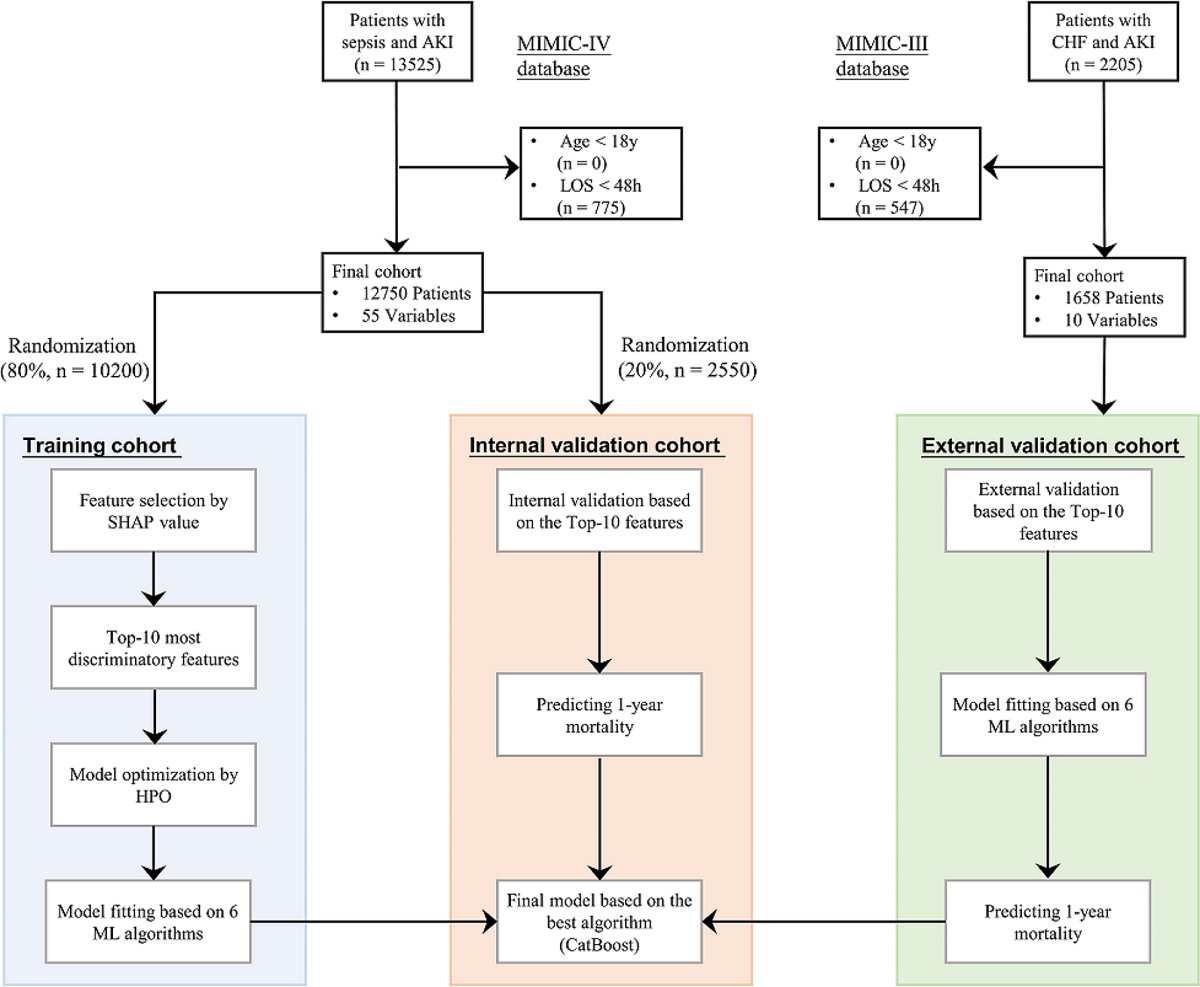
Flow chart

This study included 12,750 patients with SA-AKI, with 10,200 patients (80%) in the training set and 2550 patients (20%) in the internal validation set (Fig. 1). A total of 2442 patients (19.2%) died within the 1-year follow up period. Compared with the survival group, patients in the non-survival group were older (71.5 ± 14.7 vs. 68.0 ± 15.4, *p* < 0.001), had longer ICU stay (7.19 ± 7.98 vs. 4.91 ± 6.43, *p* < 0.001) and lower GCS score (10.0 ± 4.5 vs. 12.2 ± 3.6, *p* < 0.001). Moreover, non-survivors had more complex comorbidities and worse renal function than survivors. The baseline characteristics of the validation cohort were summarized in Table 1.

**Table 1:** Baseline characteristic

Variables	Training cohort (*n* = 12,750)			Validation cohort (*n* = 1658)		
	Survival (*n* = 10,308)	Non-Survival (*n* = 2442)	*P* value	Survival (*n* = 851)	Non-Survival (*n* = 807)	*P* value
Age, year	68.0 ± 15.4	71.5 ± 14.7	< 0.001	65.9 ± 16.8	72.0 ± 14.9	< 0.001
LOS_ICU, day	4.91 ± 6.43	7.19 ± 7.98	< 0.001	4.17 ± 6.78	6.60 ± 8.32	< 0.001
GCS score	12.2 ± 3.6	10.0 ± 4.5	< 0.001	13.6 ± 2.6	12.7 ± 3.5	< 0.001
HTN, %	7147 (69.3%)	1943 (79.5%)	< 0.001	354 (41.6%)	303 (37.5%)	0.092
CKD, %	5000 (48.5%)	1589 (65.1%)	< 0.001	103 (12.1%)	208 (25.7%)	< 0.001
HGB, g/dL	11.3 ± 2.0	10.7 ± 2.2	< 0.001	10.5 ± 1.8	10.1 ± 2.1	< 0.001
CRE, mmol/L	1.59 ± 1.57	1.80 ± 1.59	< 0.001	1.98 ± 1.74	2.20 ± 1.48	< 0.001
BUN, mmol/L	28.2 ± 19.5	37.7 ± 24.6	< 0.001	38.3 ± 25.7	47.5 ± 27.8	< 0.001
AST, U/L	103 ± 299	175 ± 407	< 0.001	425 ± 707	661 ± 887	< 0.001
UO, mL/kg/h	0.88 ± 0.64	0.66 ± 0.55	< 0.001	0.95 ± 0.72	0.60 ± 0.57	< 0.001

LOS_ICU: length of the stay in intensive care unit; GCS: Glasgow coma scale; HTN: hypertension; CKD: chronic kidney disease; HGB: hemoglobin; CRE: creatinine; BUN: blood urea nitrogen; AST: aspartate aminotransferase; UO: urine output

### Development of the prediction model

**Fig. 2:**
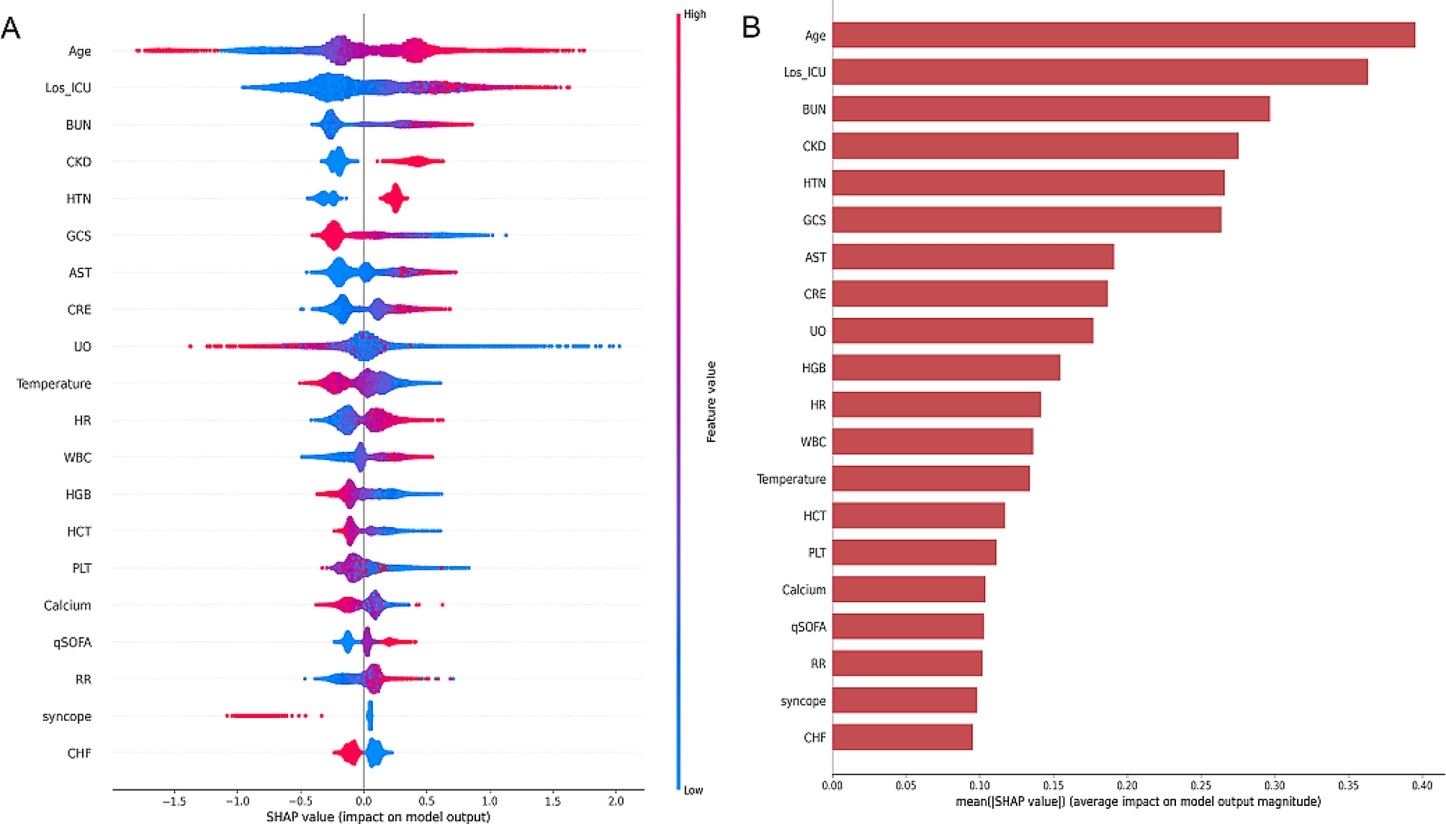
Feature Selection. (**A**) Feature importance assessed by SHAP values; the blue to red color represents the feature value (red high, blue low); the x-axis measures the impacts on the model output (right positive, left negative); (**B**) Importance of the predictors based on SHAP values

A total of 55 variables were included in this study (Table S1). To eliminate redundant or irrelevant features and improve the practicability of the model, feature selection was firstly conducted. We used the SHAP value to evaluate feature importance of all variables. The results showed that age, ICU stay, Glasgow Coma Scale (GCS) score, hypertension (HTN), chronic kidney disease (CKD), CRE, blood urea nitrogen (BUN), aspartate aminotransferase (AST), hemoglobin (HGB) and UO were the top 10 important features (Fig. 2). In this study, we only selected the top 10 features to build the prediction model. The Logistic analyses showed that the 10 features were independent risk factors for 1-year mortality in patients with SA-AKI (Table S2). In addition, SHAP force plot enabled personalized interpretation of the model (Fig. S1). To assess the multicollinearity between the 10 features, VIF test was conducted in this study. The result showed that VIFs of the 10 variables were less than 4.0, with mean VIF of 1.29, suggesting there was no significant multicollinearity between them.

**Fig. 3:**
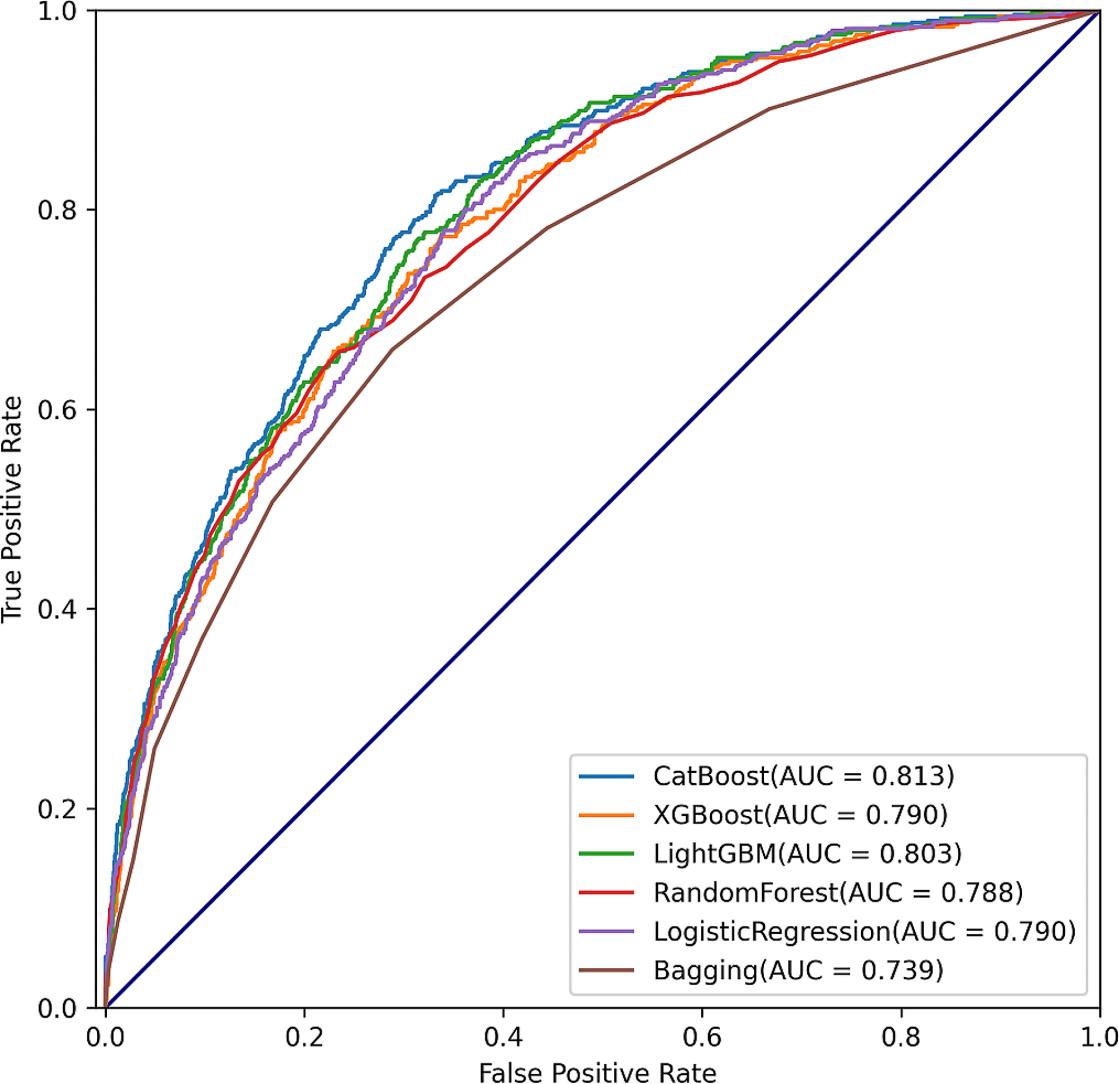
Prediction performance of different models

Based on the 10 variables, we compared the initial prediction performance (without model optimization) of the 6 ML algorithms. The result showed that the CatBoost algorithm presented with the best prediction performance with AUROC of 0.813 (Fig. 3). Furthermore, commonly used prediction model evaluation indicators, including accuracy, sensitivity and specificity, were employed to quantitative evaluation the prediction performance. We found that the CatBoost algorithm showed the best accuracy (0.833), MCC (0.646) and F1-score (0.756). The XGBoost had the best sensitivity (0.678) and NPV (0.819). And the Random Forest showed the best specificity (0.958) and PPV (0.905) (Table 2). In addition, calibration reflects the extent to which the predicted probabilities and actual probabilities agree, and is quantitively and quantitatively evaluated through calibration curve and Brier score, respectively. Brier score is calculated based on the Euclidean distance between the actual outcome and the predicted probability assigned to the outcome for each observation, with low values being desirable. In the calibration analysis, we found the prediction probability of the CatBoost model was the closest to the true probability among the 6 ML algorithms, and the CatBoost model had the lowest Brier score (0.119) (Fig. S2). Moreover, DCA was conducted to assess the clinical decision benefit which based on the prediction model by calculating the ‘net benefit’. The result showed that the CatBoost algorithm presented with the best clinical decision benefit (Fig. S3). Considering the superiority of the CatBoost algorithm in several aspects, the CatBoost was selected as the primary algorithm for remain analyses.

**Table 2:** Model performance

Model	AUROC	ACC	SENS	SPEC	PPV	NPV	MCC	F1-score
**Internal validation**								
CatBoost	**0.813**	**0.833**	0.668	0.938	0.871	0.818	**0.646**	**0.756**
LightGBM	0.803	0.830	0.666	0.932	0.861	0.816	0.638	0.752
XGBoost	0.790	0.826	**0.678**	0.920	0.842	**0.819**	0.629	0.751
Random Forest	0.788	0.822	0.634	**0.958**	**0.905**	0.806	0.639	0.746
Logistic Regression	0.790	0.810	0.621	0.929	0.846	0.796	0.594	0.716
Bagging	0.739	0.771	0.578	0.873	0.708	0.796	0.477	0.636
**External validation**								
CatBoost	**0.784**	**0.788**	0.698	0.952	**0.964**	0.634	**0.623**	0.809
LightGBM	0.772	0.786	0.724	0.899	0.929	0.641	0.596	0.812
XGBoost	0.747	0.779	**0.743**	0.846	0.898	0.644	0.565	**0.813**
Random Forest	0.758	0.784	0.691	**0.952**	0.963	0.628	0.617	0.805
Logistic Regression	0.767	0.781	0.724	0.887	0.921	0.638	0.585	0.810
Bagging	0.682	0.761	0.670	0.913	0.901	**0.701**	0.593	0.768

AUROC: area under the receiver operating characteristic curve; ACC: accuracy; SENS: sensitivity; SPEC: specificity; PPV: positive prediction value; NPV: negative prediction model; MCC: Matthews correlation coefficient

Algorithm optimization is also a pivotal procedure for ML-based prediction model building. In the present study, HPO was employed to improve the performance of the CatBoost model. Based on the Optuna framework, a total of 100 trials of optimal hyperparameter searching were performed, as a result, the best combination of hyperparameters was obtained (Fig. S4). The hyperparameter search domains and final settings were listed in Table S3. After HPO, the prediction performance of the CatBoost was significantly improved with an AUROC of 0.837 (Fig. S5). Based on the optimized CatBoost model, we discovered that the model could accurately predict 1-year mortality of both early and late SA-AKI, achieving AUROCs of 0.848 and 0.805, respectively (Fig. S6).

### Model evaluation and validation

To further demonstrate the prediction performance of the CatBoost model, the CatBoost model was compared with other commonly used clinical scores, including LODS, SAPS and Charson comorbidity index, which could evaluate the condition severity generally. The result showed that the CatBoost model presented with the best prediction performance (Fig. S7). In addition, the external validation was performed to demonstrate the generalizability of the CatBoost model. We extracted data of the 10 features and the outcome from the MIMIC-III database to perform the temporal external validation. The results showed that the CatBoost model presented with the best prediction performance (Fig. S8), with AUROC, accuracy, sensitivity, specificity and F1-score of 0.788, 0.698, 0.952 and 0.809, respectively (Table 2). Accordingly, we suggested that the CatBoost model had the certain generalizability.

### Risk stratification

**Fig. 4:**
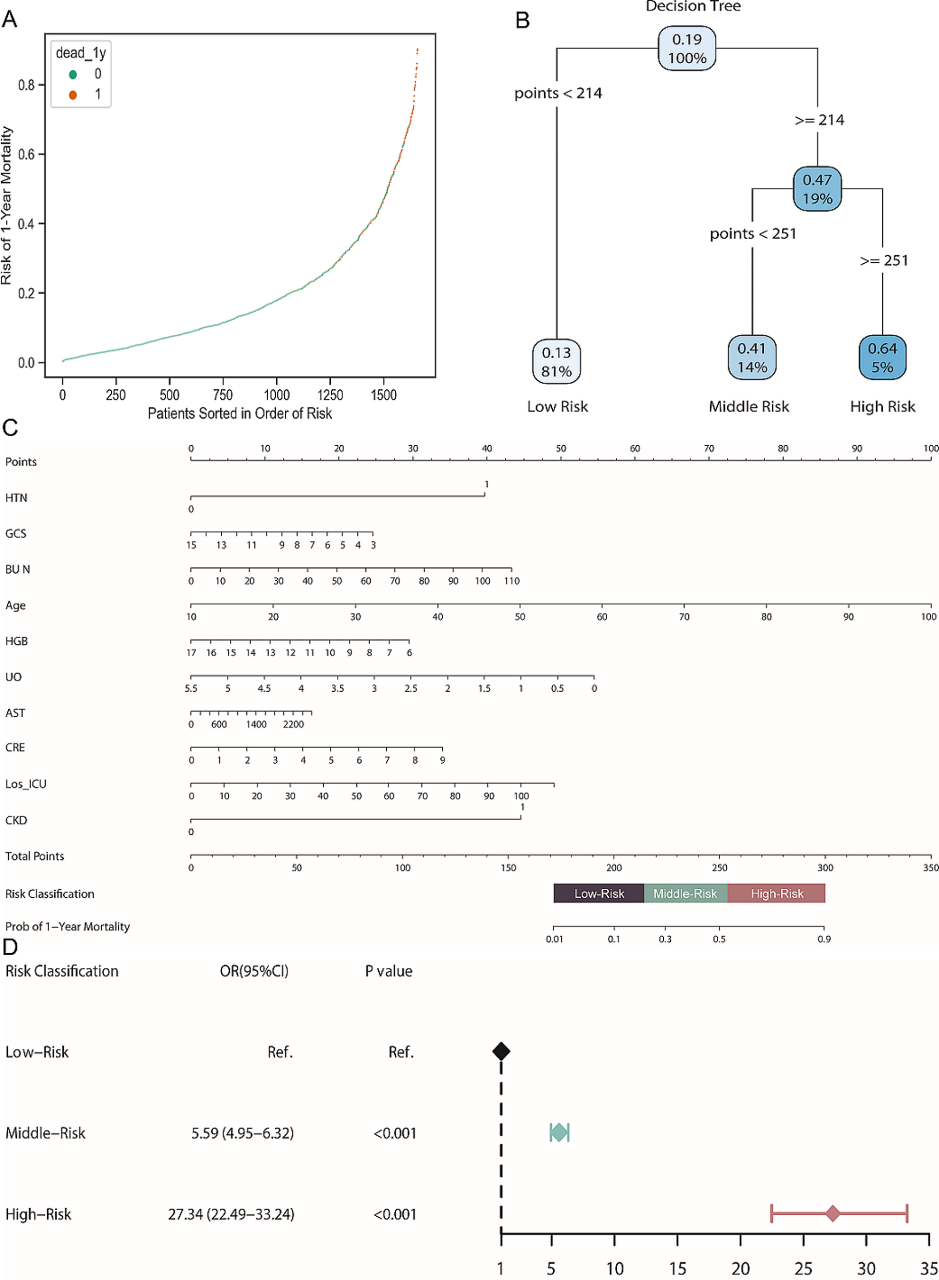
Model validation and risk stratification. (**A**) Prediction distributions of the risk of 1-year mortality; (**B**) The risk stratification of 1-year mortality according to a decision tree; (**C**) A nomogram of the CatBoost model for predicting 1-year mortality during in patients with sepsis associated acute kidney injury; (**D**) Logistic analysis of the risk of 1-year mortality based on the risk stratification

Risk stratification enabled early identification of high-risk patients at poor prognosis and subsequently personalized clinical decision-making. In this study, we developed a personalized nomogram and a risk stratification tool to elucidate the practicability of the CatBoost model. First, the 1-year mortality probabilities of each patient in the external cohort were obtained using the *‘predict_proba’* function of the CatBoost algorithm. The patients were ranked by the prediction probability. The prediction distribution plot of the CatBoost model with patients sorted in the order of risk showed positive clustering of patients who died within the 1-year follow up, suggesting the favorable discriminatory ability of the model (Fig. 4A). Second, the decision tree algorithm was employed to realize the risk stratification using the ‘*rpart.plot*’ package (Fig. 4B). Third, a nomogram based on the 10 features was developed. The total points of each patient were calculated using the ‘*nomogramFormula*’ package. Then two cut-off values based on the total point were obtained using the decision tree algorithm. Accordingly, patients were divided into three groups: low-risk (total points < 214), middle-risk (total points ≥ 214 and < 251), and high-risk group (total points ≥ 251) (Fig. 4C). Final, the Logistic analysis was conducted to demonstrate the risk stratification ability of the nomogram. Compared with the low-risk group, patients in the middle- and high-risk group had a 5-fold and 27-fold risk of 1-year mortality, respectively (Fig. 4D), which suggested the favorable practicability of the CatBoost model.

## Discussion

In this study, we developed and validated an ML-based model to accurately predict 1-year mortality of patients with SA-AKI using 6 commonly used ML algorithms. We screened 10 key features, including age, ICU stay, GCS score, HTN, CKD, HGB, CRE, BUN, AST and UO, to build the prediction model. Our model showed superior prediction performance than traditional risk scores, including LODS, SAPS-II and Charson comorbidity index. The favorable performance was also validated in the external validation set. The prediction model enabled early identification of SA-AKI patients with high-risk of poor prognosis, which may help to optimize the management of the patients with SA-AKI and to improve the outcomes.

All six ML classifiers included in this study were fully established and commonly used to perform prediction issues. Although the difference of prediction performance between the 6 ML algorithms was insignificant, the CatBoost algorithm presented with the best performance among them. The CatBoost belongs to gradient boosting algorithms and could successfully handle categorical features and takes advantage of dealing with them during training as opposed to preprocessing time. Moreover, CatBoost uses a new schema to calculate leaf values when selecting the tree structure, which is conducive to reducing overfitting [25]. The superiority of the CatBoost algorithm has been demonstrated in our previous study [12].

Although the typical advantage of using ML models is that they can handle higher dimensional data, numerous variables would reduce the practicability of the model. Therefore, we sought to develop a refined model based on the top predictors. A total of 10 key features (age, ICU stay, GCS score, HTN, CKD, HGB, CRE, BUN, AST and UO) were identified by using the SHAP value. The CatBoost model also showed the satisfactory prediction performance only based on the 10 variables. Among the 10 key features, age played the most important role in the model of predicting 1-year mortality.

In general, elders had more comorbidities than young patients, thus had a poorer outcome. Hu et al. also found that age was an independent risk factor for short-term mortality in patients with SA-AKI [26]. Longer ICU stay and lower GCS score represented the more severe condition of patients. The predictive values of ICU stay and GCS score in predicting prognosis in SA-AKI patients have been demonstrated in the previous study [27]. CKD, CRE, BUN and UO were highly related to renal function, and were predictors for mortality in patients with SA-AKI [27].

Because both sepsis and AKI are clinical diagnoses, it is difficult to identify the exact onset of organ injury. Accordingly, risk stratification tool and clinical risk scores are important for decision-making in patients with SA-AKI. There were several clinical scores, such as LODS and SAPS-II score, which were widely used to predict outcomes in the critical care settings. However, these clinical scores were limited by the undistinguished prediction performance and inadequate specificity [28, 29]. In the present study, the performances of the LODS and SAPS-II in predicting 1-year mortality in SA-AKI patients were common, with AUROC of 0.719 and 0.703, respectively. Moreover, there were very limited prediction models to predict the long-term prognosis of patients with SA-AKI. Hu et al. [26] and Luo et al. [27] only focused on the development of clinical models to predict in-hospital and short-term mortality in patients with SA-AKI. In this study, we firstly built a clinical model that enabled accurate prediction of 1-year mortality in SA-AKI patients.

It is the determining factor for a clinical application whether a model has practicality. The previous studies mainly concentrate on the model development itself, but little on the application value [9, 26, 27]. In this study, we established a risk stratification tool based on the nomogram that enabled easily and accurately identification of SA-AKI patients with high-risk of poor prognosis. Moreover, the 10 features for the nomogram development were readily accessible and frequently monitored in routine clinical practice, therefore, the model could be generalized on a large scale, especially for undeveloped regions.

### Limitations

Our study has several limitations. First, our work is based on a retrospective analysis of data, and further prospective studies are needed to confirm the findings. Second, data used in this study are extracted from public databases. Many important variables including C-reaction protein and procalcitonin are excluded for the unacceptable rate of missing values, which may affect the final model. Additionally, the absence of follow-up data limits our models’ ability to predict major adverse kidney events effectively. Third, treatments including antibiotics, vasoactive agents, or mechanical ventilation are not included in this study for inadequate data, which may provide some biases.

## Conclusion

In the present study, we developed and validated an ML-based model which could accurately predict for 1-year mortality in patients with SA-AKI. Moreover, we established a risk stratification tool based on the 10 key features of the nomogram that enabled early identification of high-risk patients, thus, prognosis could be improved by providing reasonable alerting and feedback.

### Electronic supplementary material

Below is the link to the electronic supplementary material.

Supplementary Material 1

## Data Availability

Publicly available datasets were analyzed in this study. The data from the Medical Information Mart for Intensive Care IV (MIMIC-IV, version 2.0) and the MIMIC-III (version 1.4) can be found at https://physionet.org/content/mimiciv/2.2 and https://physionet.org/content/mimiciii/1.4.
