# Supplementary material for: Machine learning for the prediction of 1-year mortality in patients with sepsis-associated acute kidney injury
Source: BMC Med Inform Decis Mak. 2024 Jul 25;24:208. doi: 10.1186/s12911-024-02583-3 (PMC11271185; doi:10.1186/s12911-024-02583-3)
Supplement: Supplementary file 1 — Supplementary Material 1 [file 12911_2024_2583_MOESM1_ESM.docx]

**Supplementary Online Content**

**Table S1.** Total 55 predictor variables used in machine learning models

**Table S2**. Univariate and multivariate logistic regression analyses for 1-year mortality

**Table S3.** Hyperparameter search domains and final settings

**Figure S1.** SHAP force plot

**Figure S2**. Calibration curve of the six machine learning models.

**Figure S3**. Decision curve analysis of the six machine learning models.

**Figure S4**. Hyperparameter optimization

**Figure S5**. The prediction performance of CatBoost model before and after HPO.

**Figure S6.** The prediction performance of CatBoost model after HPO for predicting 1-year mortality of (A) early SA-AKI and (B) late SA-AKI.

**Figure S7**. Prediction performance comparisons between the CatBoost model and other commonly used clinical scores.

**Figure S8**. External Validation.

| **Table S1.** Total 55 Predictor Variables Used in Machine Learning Models | | | | |
| --- | --- | --- | --- | --- |
| Age | DBP | CHF | VA | BUN |
| Male | RR | Valvular disease | Syncope | ALT |
| Weight | SpO_2_ | Dyslipidemia | RRT | AST |
| LOS | UO | CKD | WBC | Glucose |
| GCS score | Diabetes | Hepatitis | RBC | Sodium |
| SOFA score | PE | Liver cirrhosis | PLT | Potassium |
| Race | Hypertension | Stroke | Hemoglobin | Calcium |
| Temperature | AF | COPD | Hematocrit | Chloride |
| HR | AMI | Pneumonia | SCr | Phosphorus |
| MAP | OMI | Cancer | INR | Magnesium |
| SBP | Angina pectoris | Anemia | PT | qSOFA |

LOS: total length of stay in hospital prior to acute kidney injury; GCS: Glasgow coma scale; SOFA: Sequential Organ Failure Assessment; HR: heart rate; MAP: mean aortic pressure; SBP: systolic blood pressure; DBP: diastolic blood pressure; RR: respiratory rate; SpO2: saturation of pulse oxygen; UO: urine output; PE: pulmonary embolism; AF: atrial fibrillation; AMI: acute myocardial infarction; OMI: old myocardial infarction; CKD: chronic kidney disease; COPD: chronic obstructive pulmonary disease; VA: ventricular arrhythmia (ventricular tachycardia, ventricular fibrillation); RRT: renal replacement therapy; WBC: white blood cell count; RBC: red blood cell count; PLT: platelet count; SCr: Serum creatinine; INR: international normalized ratio; PT: prothrombin time; BUN: blood urine nitrogen; ALT: alanine transaminase; AST: aspartate transaminase; qSOFA: quick Sequential Organ Failure Assessment.

| **Table S2.** Univariate and multivariate logistic regression analyses for 1-year mortality. | | | | | | | |
| --- | --- | --- | --- | --- | --- | --- | --- |
|  | Univariate logistic analysis | |  | | Multivariate logistic analysis | | |
| **Variables** | OR (95% CI) | P value | |  | | OR (95% CI) | P value |
| Age | 1.014 (1.011–1.017) | < 0.001 | |  | | 1.020 (1.017–1.024) | < 0.001 |
| LOS_ICU | 1.031 (1.026–1.037) | < 0.001 | |  | | 1.055 (1.014–1.061) | < 0.001 |
| GCS score | 0.885 (0.877–0.894) | < 0.001 | |  | | 0.899 (0.888–0.909) | < 0.001 |
| HTN | 2.312 (2.214–2.516) | < 0.001 | |  | | 2.562 (2.323–2.826) | < 0.001 |
| CKD | 1.855 (1.700–2.023) | < 0.001 | |  | | 3.335 (2.991–3.719) | < 0.001 |
| HGB | 0.868 (0.851–0.886) | < 0.001 | |  | | 0.874 (0.854–0.894) | < 0.001 |
| CRE | 1.104 (1.078–1.130) | < 0.001 | |  | | 1.054 (1.017–1.094) | < 0.001 |
| BUN | 1.020 (1.018–1.022) | < 0.001 | |  | | 1.023 (1.020–1.026) | < 0.001 |
| AST | 1.007 (1.006–1.008) | < 0.001 | |  | | 1.005 (1.004–1.006) | < 0.001 |
| UO | 0.468 (0.431–0.509) | < 0.001 | |  | | 0.520 (0.476–0.567) | < 0.001 |

OR: odd ratio; other abbreviations were the same as in eTable 1.

**Table S3**. Hyperparameter Search Domains and Final Settings

| Hyperparameters | Type | Search domain | Final setting |
| --- | --- | --- | --- |
| 'depth' | Choice | {4, 5, 6, 7, 8} | 6 |
| 'bagging_temperature' | Uniform | [0, 5] | 3.1407 |
| 'reg_lambda' | Uniform | [1, 5] | 1.7985 |
| 'learning_rate' | Uniform | [0.01, 0.05] | 0.0109 |
| 'min_data_in_leaf' | Choice | {1, 2, 3} | 2 |


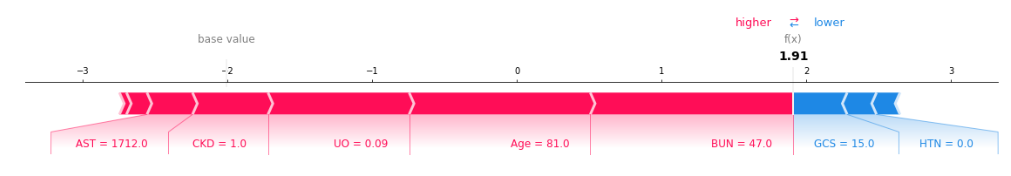
 **Figure S1**. SHAP Force Plot. This is a non-survivor at 1-year. The force plot shows each feature contributions for pushing model's prediction from base value to final output. Base value represents the average model output over the derivation cohort. Red means that the contribution of feature is positive, and blue means that the contribution of feature is negative. The length of the color bar represents the amount of contribution.


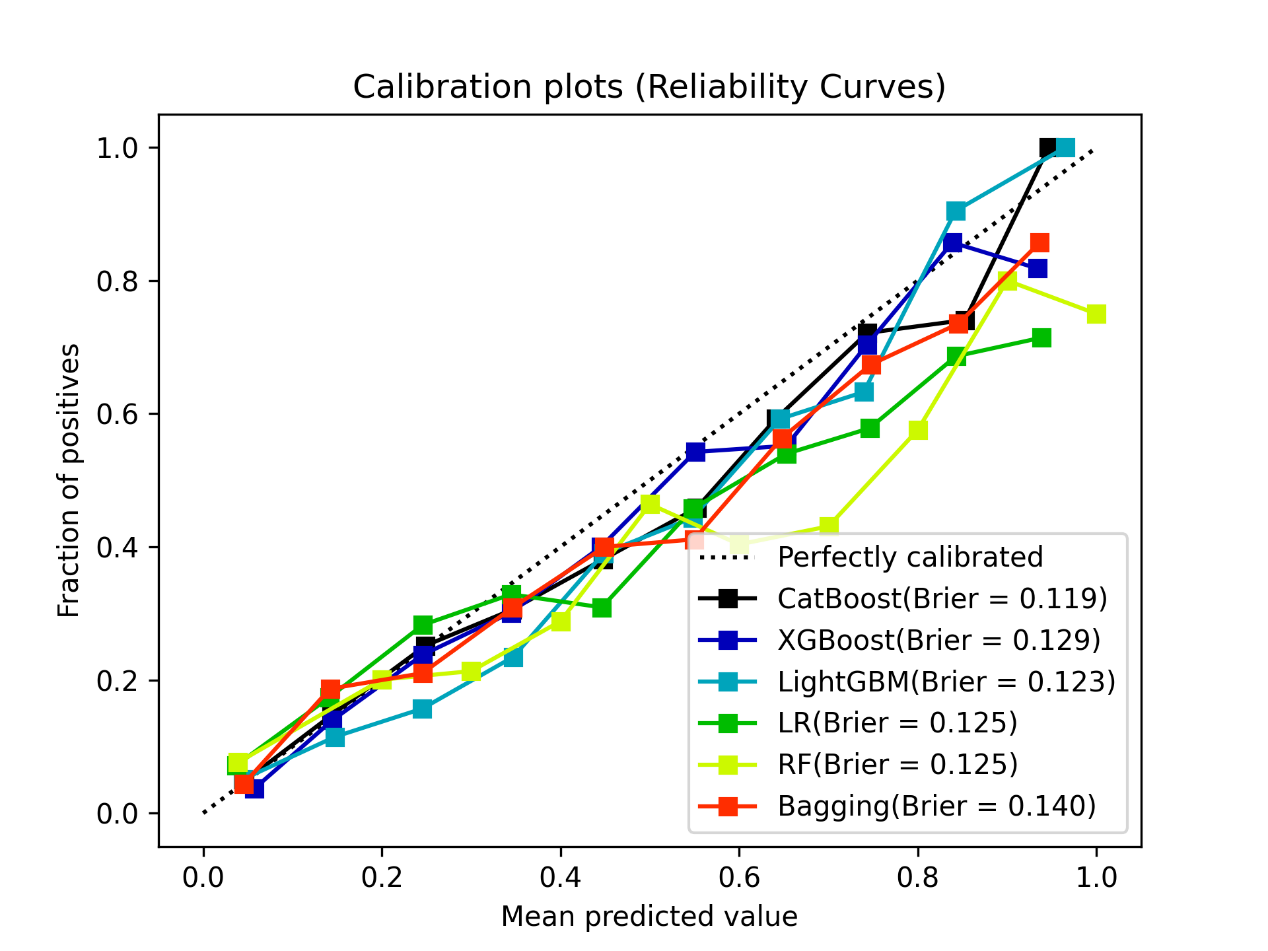


**Figure S2** Calibration curve of the six machine learning models.


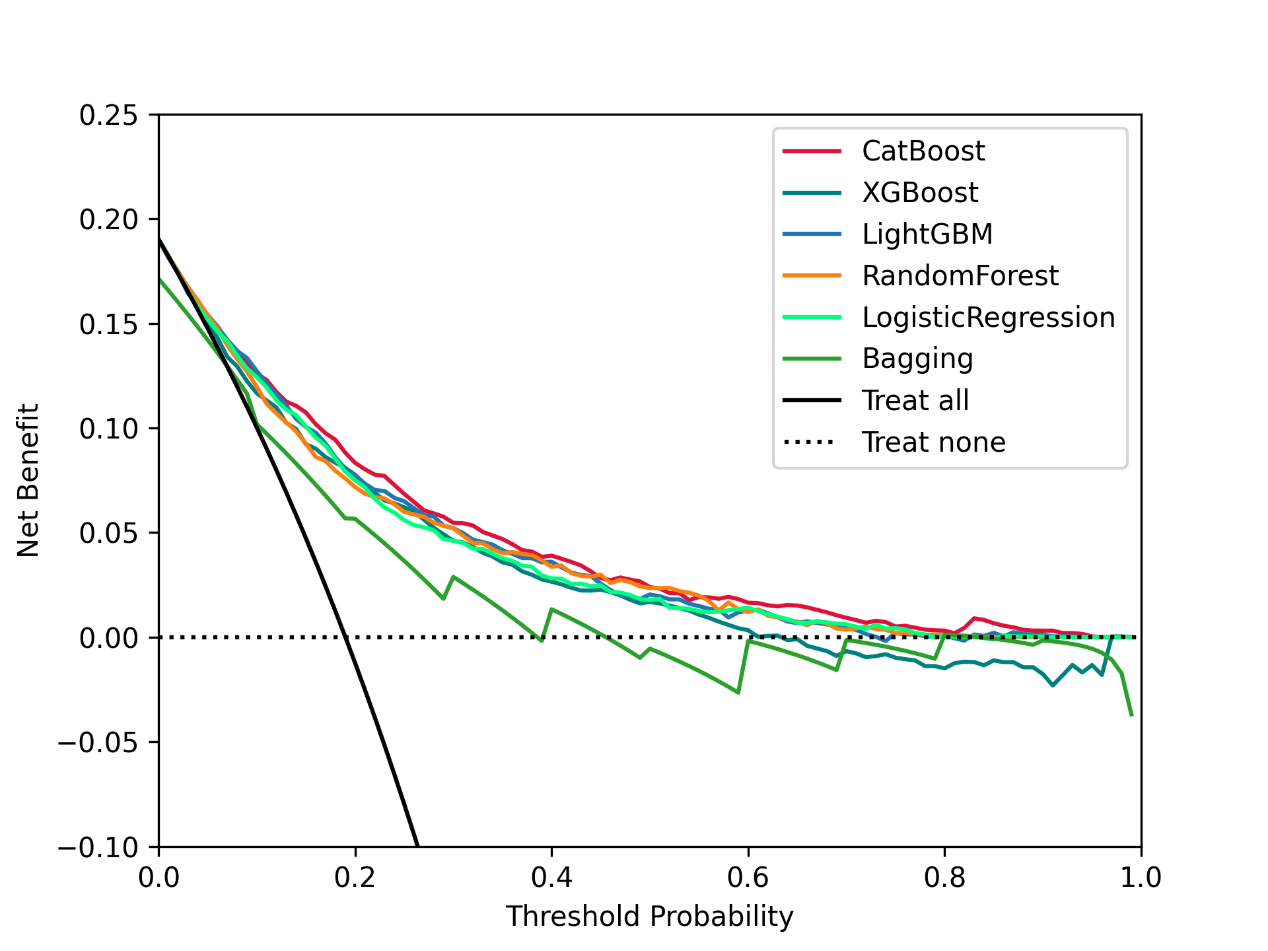


**Figure S3**. Decision curve analysis of the six machine learning models.


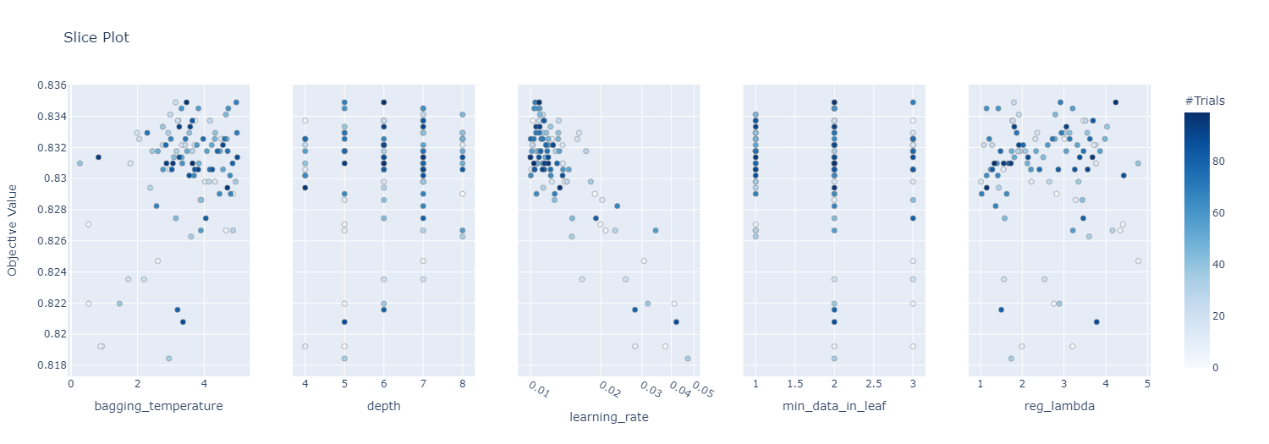

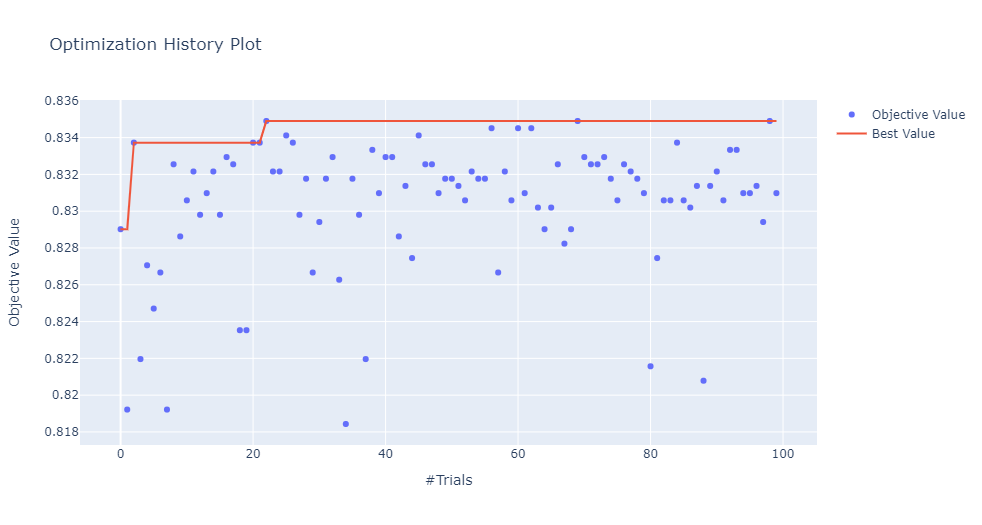

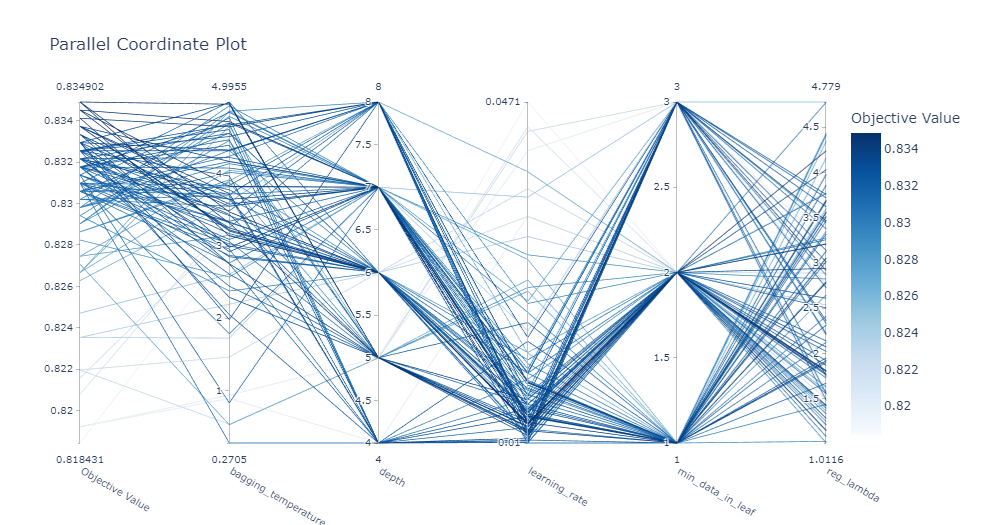
**Figure S4**. Hyperparameters optimization. (**A**) History of hyperparameters optimization; each blue point represents the result of a trial, and the dark orange line represents the best AUC value; (**B**) Parallel coordinate plot of parameter; Each line represents a trial; the shade of color represents the performance of optimization; (**C**) Slice plot of every single parameter; the shade of color represents the influence of every parameter on the model performance.

**C**

**B**

**A**


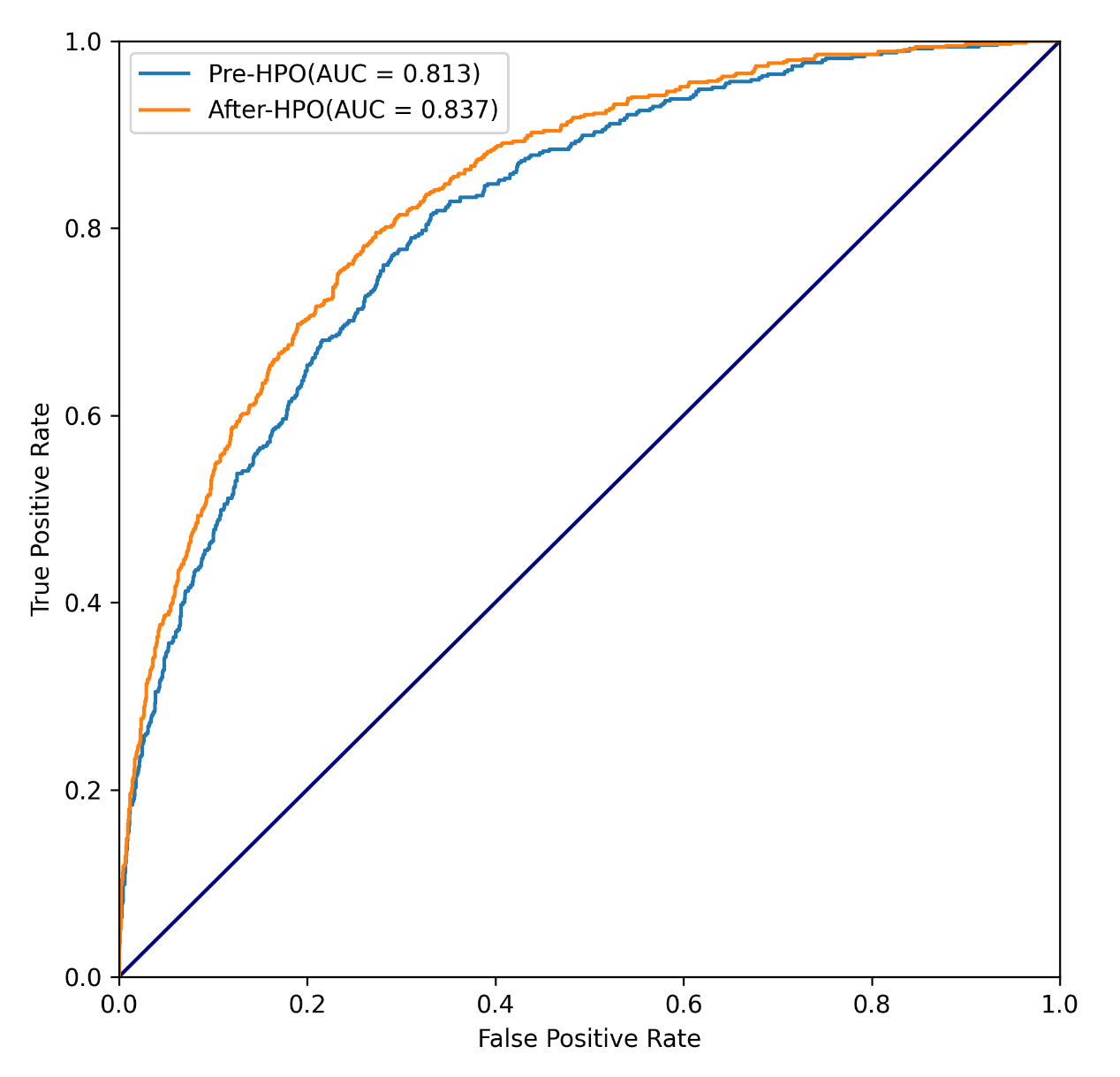


**Figure S5**. The prediction performance of CatBoost model before and after HPO.


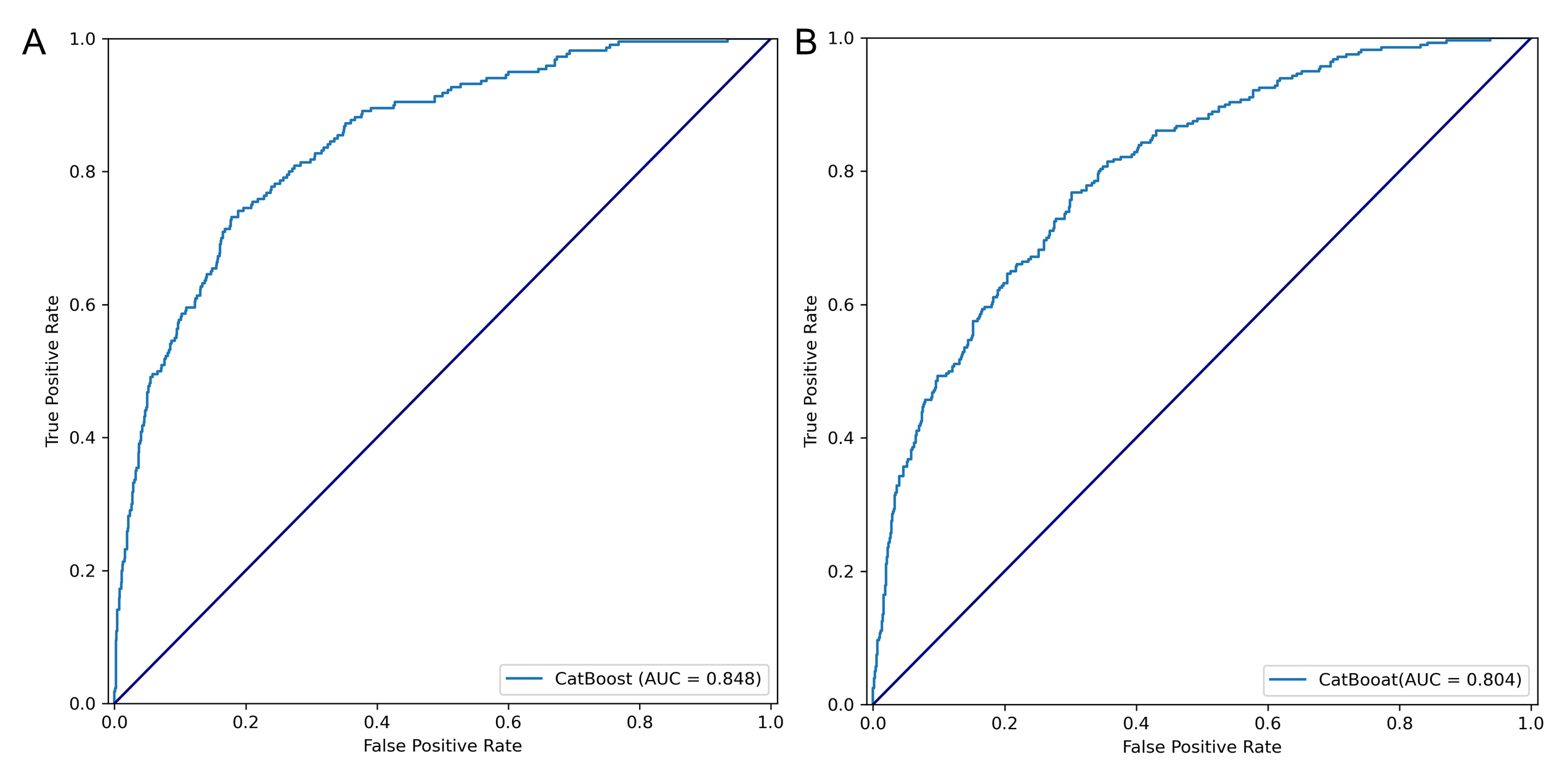


**Figure S6**. The prediction performance of CatBoost model after HPO for predicting 1-year mortality of (**A**) early SA-AKI and (**B**) late SA-AKI.


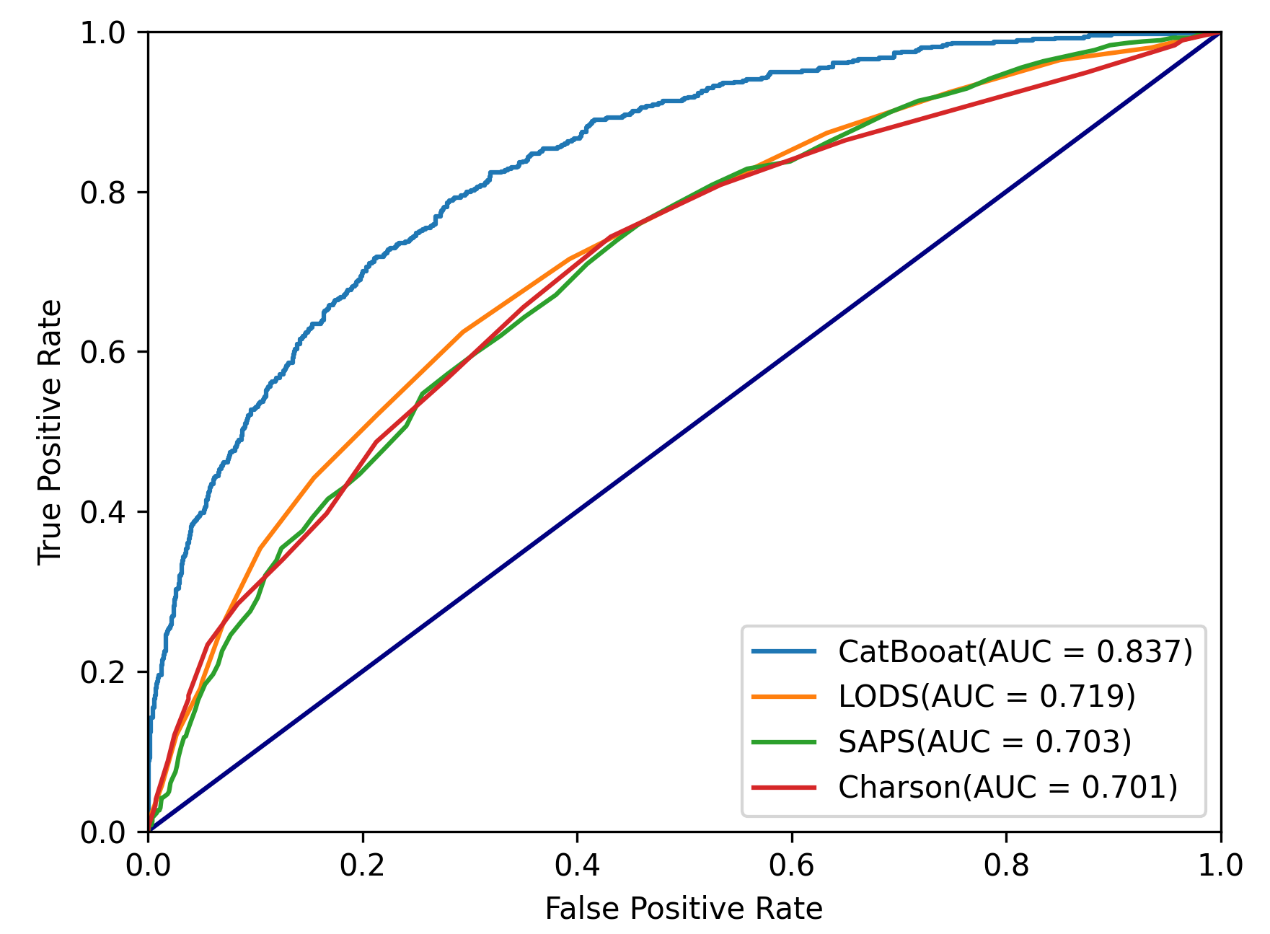


**Figure S7**. Prediction performance comparisons between the CatBoost model and other commonly used clinical scores. LODS: Logistic Organ Dysfunction System; SAPS: Simplified Acute Physiology Score-II.


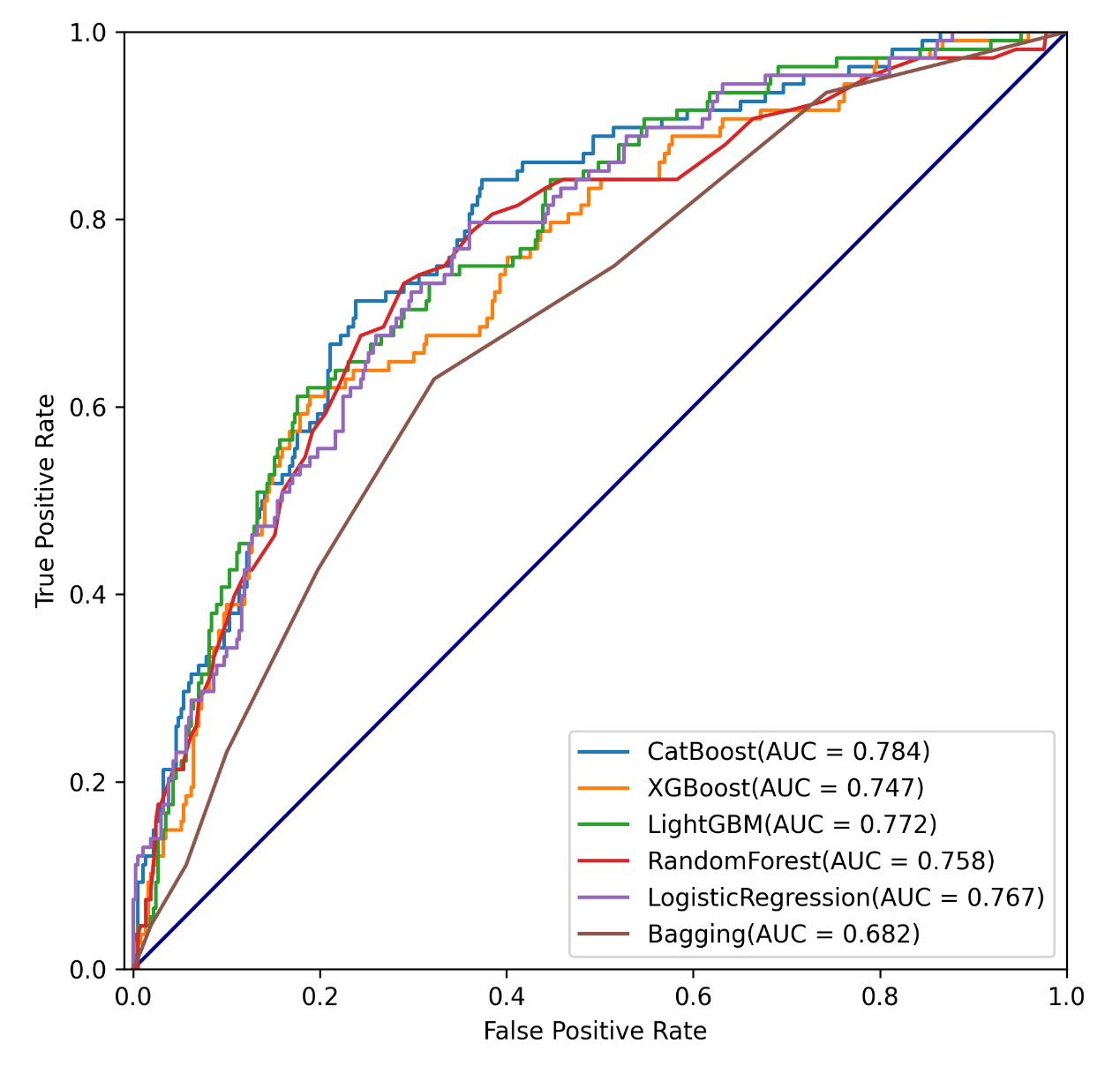


**Figure S8**. External Validation.
